# Severe Permanent Visual Decline After Hypotony-Induced Retinal Pigment Epithelial Changes

**DOI:** 10.7759/cureus.99551

**Published:** 2025-12-18

**Authors:** Konstantinos Benekos, Konstantina Gorgoli, Panagiotis Laspas, Panagiotis A Konstas, Andreas Katsanos

**Affiliations:** 1 Ophthalmology Department, University Hospital of Ioannina, Ioannina, GRC

**Keywords:** choroidal detachment, hyperautofluorescent rpe lines, hypotony, hypotony maculopathy, paul glaucoma implant, retinal pigment epithelium

## Abstract

Postoperative hypotony accompanied by choroidal effusions and maculopathy are well-recognized complications following glaucoma shunt surgery. We report a rare case of an elderly patient with irreversible visual loss in his left eye (OS) caused by retinal pigment epithelium (RPE) changes secondary to short-term hypotony maculopathy and choroidal detachment after a Paul glaucoma drainage device implantation.

An 83-year-old man presented with recurrent episodes of transient loss of vision in his OS. Examination of his OS revealed high intraocular pressure (IOP), a one-piece in-the-bag posterior chamber intraocular lens (IOL) with pseudophacodonesis and hyphema, pointing toward the diagnosis of uveitis-glaucoma-hyphema (UGH) syndrome. Despite the urgent extraction of the IOL-capsular bag complex, pars plana vitrectomy, and implantation of a retro-pupillary iris-claw lens, the IOP was poorly controlled with maximal medical treatment, necessitating the implantation of a Paul drainage device. Postoperatively, kissing choroidal detachments were observed due to early postoperative hypotony. Although the IOP soon normalized and the choroidal detachment resolved, the visual acuity remained markedly reduced due to apparently permanent hypotony-related subfoveal RPE changes that were well documented with fundus autofluorescence.

This case highlights that even a short period of hypotony compounded by choroidal detachment and hypotony maculopathy can cause permanent RPE damage and eventually irreversible visual loss. The pattern and distribution of the RPE changes were associated with the areas of prior choroidal detachment. Notably, autofluorescence proved particularly useful for the assessment of these subtle but vision-threatening RPE changes.

## Introduction

Glaucoma drainage implants are indicated when standard medical and surgical approaches, such as trabeculectomy, are likely to fail [1]. These devices reduce intraocular pressure (IOP) by draining the aqueous humor from the eye into an external reservoir. The valveless Paul glaucoma drainage device has been increasingly used in recent years for the management of elevated IOP in patients with refractory glaucoma [2]. Although glaucoma drainage implants constitute vital options in a number of patients, their use is sometimes plagued with serious complications [3].

Postoperative hypotony, often accompanied by choroidal effusions and maculopathy, is a well-recognized complication after plate-based shunt surgery, especially when non-valved implants, such as a Paul or Baerveldt device, are used. In plate-based shunt surgery, the reported rates of this complication range from 2% to 35.4% [2,4,5]. These complications are most commonly observed during the early postoperative period, before the implant endplate is adequately encapsulated [6]. Notably, the simultaneous occurrence of choroidal effusions and maculopathy has been reported to be rather uncommon, especially in the elderly [7]. While most cases with hypotony accompanied by choroidal effusions and maculopathy are self-limiting and resolve within a short period with conservative measures, long-lasting hypotony, particularly when accompanied by hypotony maculopathy, can lead to permanent central visual loss and irreversible RPE changes [8].

In this report, we present the case of a patient with permanent severe visual decline due to retinal pigment epithelium (RPE) changes as a consequence of hypotony-induced choroidal detachment and hypotony maculopathy following the implantation of a Paul drainage device. Remarkably, in our case, a relatively brief period of hypotony was sufficient to cause both choroidal effusions and hypotony maculopathy. This led to profound and apparently permanent RPE damage with severe visual loss.

## Case presentation

An 83-year-old man presented in the ophthalmic emergency service with his third episode of recurrent transient blurred vision in the left eye (OS) within the past year. For the previous two episodes, he did not seek medical assistance. His past medical history involved systemic hypertension, dyslipidemia, and benign prostatic hyperplasia. His ophthalmic history included exfoliative syndrome and ocular hypertension treated with latanoprost once per day, along with a fixed combination of dorzolamide and timolol twice daily. His OS had uncomplicated phacoemulsification with posterior chamber intraocular lens (IOL) implantation in the capsular bag five years ago. 

**Figure 1:**
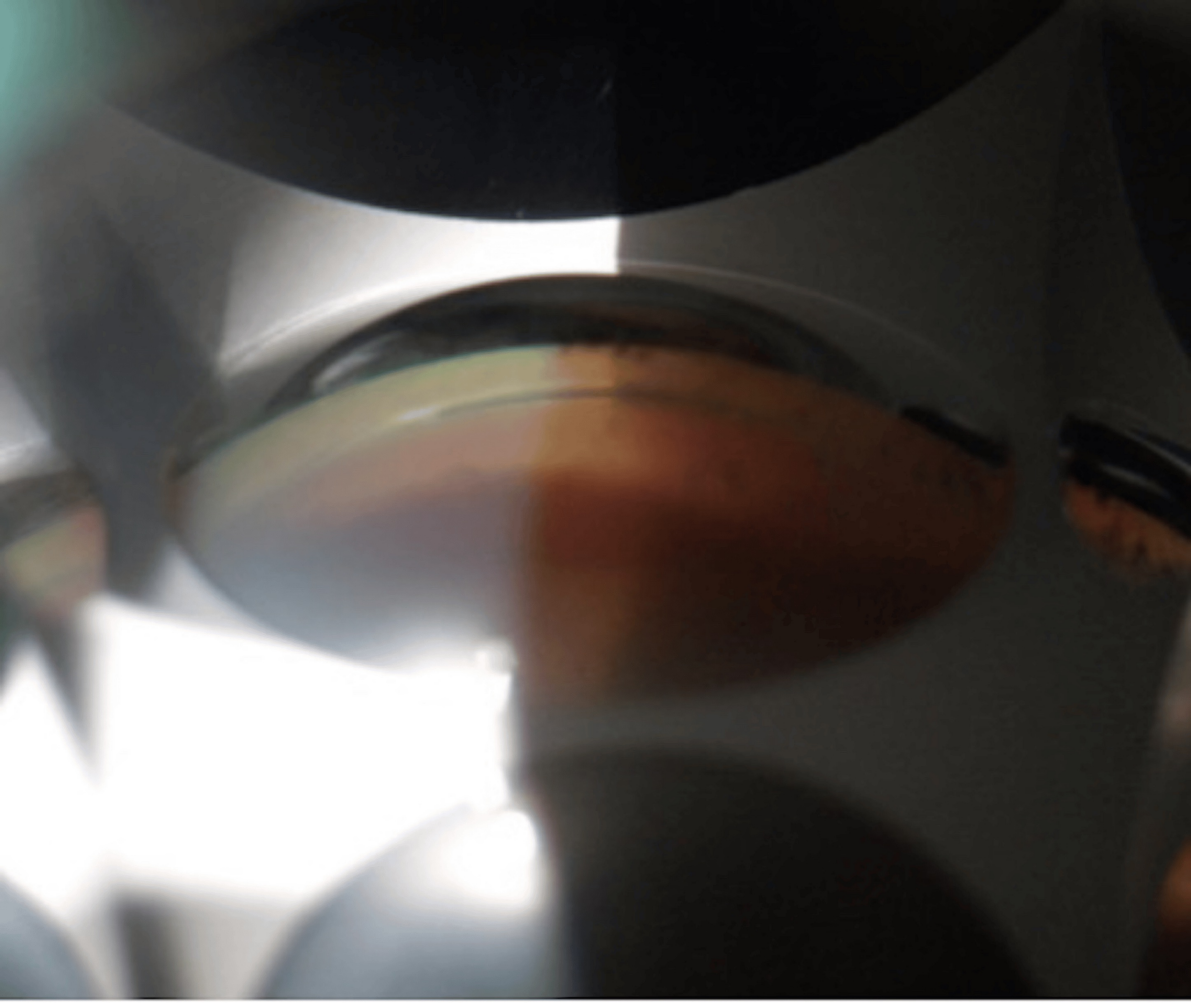
Hyphema observed in the inferior angle of the left eye

**Figure 2:**
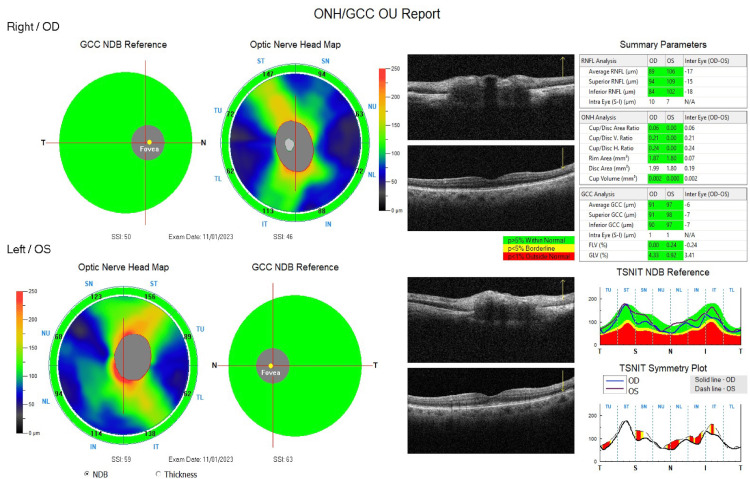
Optical coherence tomography of the retinal nerve fiber layer of both eyes within normal limits, without any glaucomatous damage

**Figure 3:**
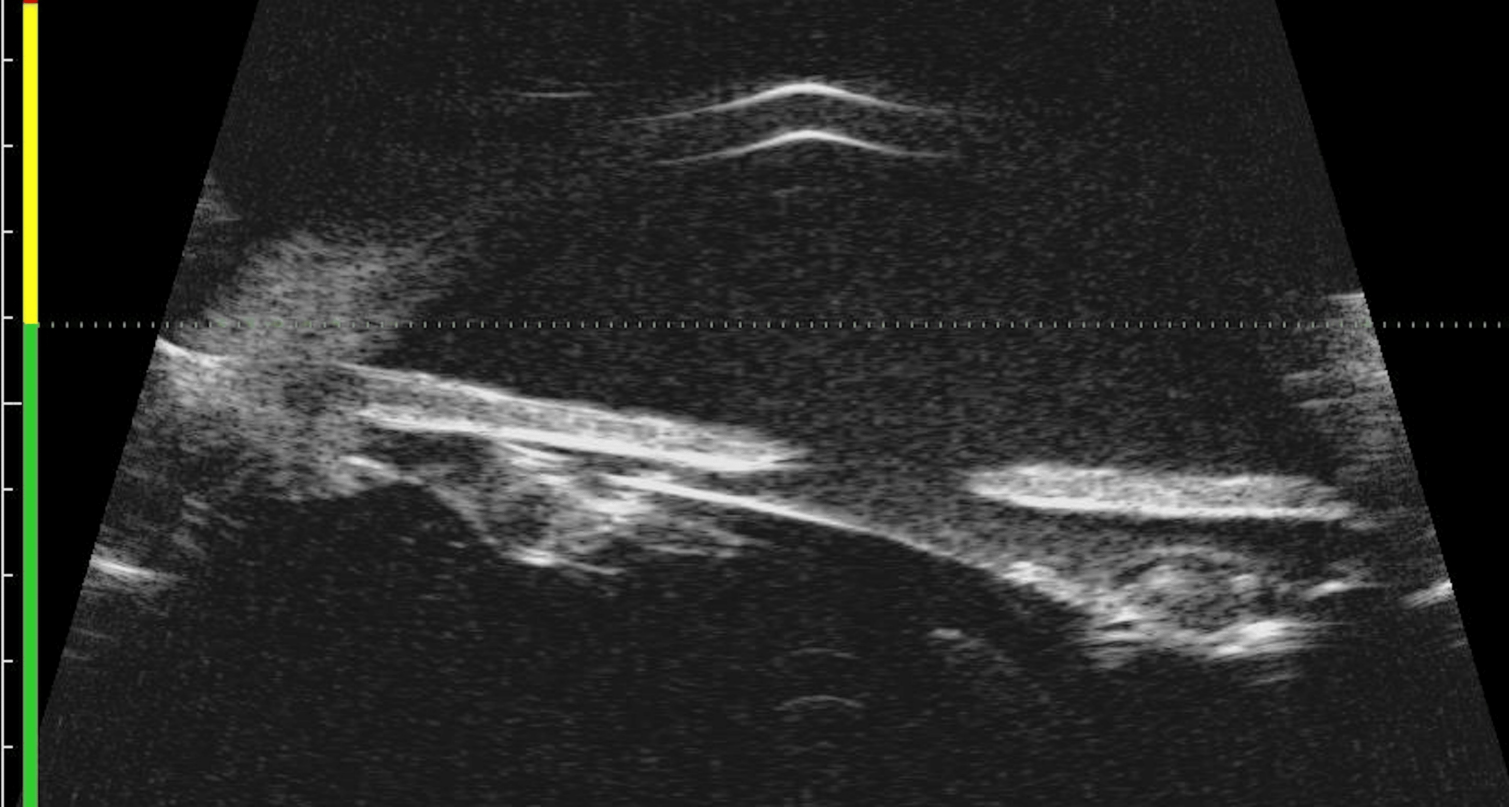
Ultrasound biomicroscopy of the left eye showing iris-intraocular lens contact

On clinical examination, distance best corrected visual acuity (BCVA) was logMAR 0.52 in the right eye (OD) and logMAR 0.40 in the OS, while the IOP was measured at 16 mmHg (OD) and 54 mmHg (OS). The slit-lamp examination in the OS revealed a one-piece in-the-bag posterior chamber IOL with pseudophacodonesis, exfoliative material on the pupillary border with absence of any iris neovascularization or transillumination, and hyphema < 1 mm, accompanied by scattered red blood cells in the anterior chamber (Figure 1). The examination in the OD was unremarkable, with the exception of nuclear and cortical cataracts. In fundoscopy, a small parafoveal choroidal nevus of the left fundus was observed, with no evidence of glaucomatous optic nerve damage in any eye (Figure 2). Ultrasound biomicroscopy in the OS detected anterior dislocation of the IOL-capsular bag complex with iris-IOL contact, likely secondary to the exfoliation syndrome (Figure 3), pointing toward the diagnosis of the uveitis-glaucoma-hyphema (UGH) syndrome. The patient’s treatment was escalated with the addition of acetazolamide 125 mg every eight hours per os and topical dexamethasone drops five times per day. Urgent extraction of the IOL-capsular bag complex, pars plana vitrectomy, and implantation of a retro-pupillary iris-claw lens were performed. On the day of the surgery, the preoperative IOP of the OS was 32 mmHg, while BCVA was stable at logMAR 0.40, and the IOL-capsular bag complex was still in place.

The surgery was completed without any intraoperative complications. On the first postoperative day, the IOP was 26 mmHg, and BCVA was hand movement. Despite the complete resolution of the signs of the UGH syndrome and BCVA of logMAR 0.7, 20 days after surgery, the IOP remained uncontrolled and increased to approximately 40 mmHg on maximal medical treatment. Therefore, the placement of a Paul drainage device was undertaken 10 days later. 

One day before the tube shunt surgery, the clinical examination revealed no unknown abnormalities, and the IOP and BCVA of the OS were again 40 mmHg with maximum medical treatment and logMAR 0.7, respectively, with the iris-claw lens well placed behind the iris. The Paul implant was placed on the upper temporal quadrant between the superior and lateral rectus muscles, and one Vicryl 7.0 strangulation suture was placed around the tube so as to reduce the aqueous flow. No viscoelastic was placed in the anterior chamber during the operation, and a scleral patch was utilized to prevent conjunctival erosion. On the first postoperative day, the IOP was equal to 2 mmHg, with a deep anterior chamber along with Descemet membrane folds and corneal edema, while kissing choroidals were observed after seven days. The hypotony and choroidal effusions were treated conservatively with topical atropine instillation along with topical dexamethasone.

**Figure 4:**
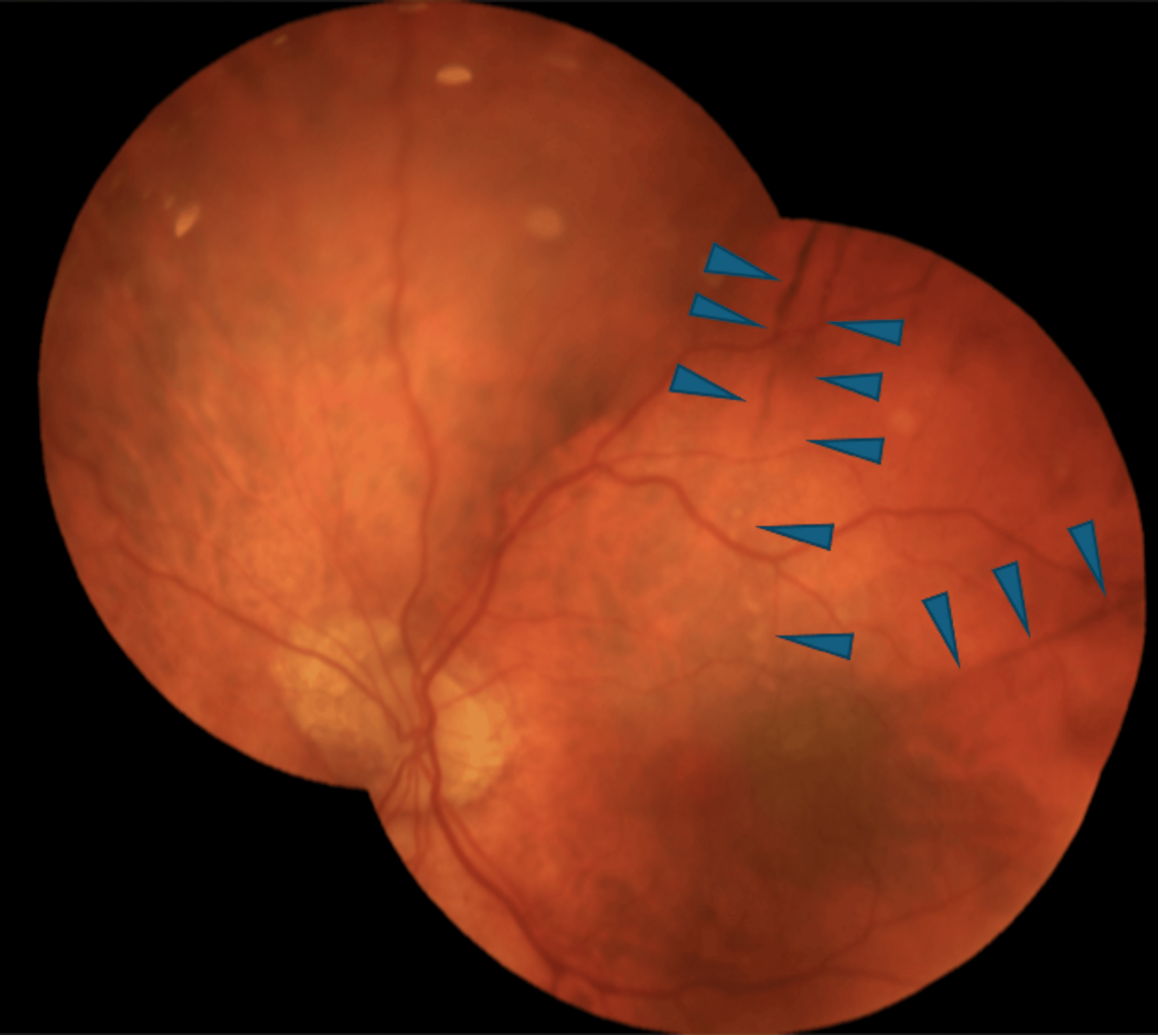
Fundus photograph of the left eye after choroidal detachment remission. Arrowheads point to the most prominent retinal pigment epithelium lines

**Figure 5:**
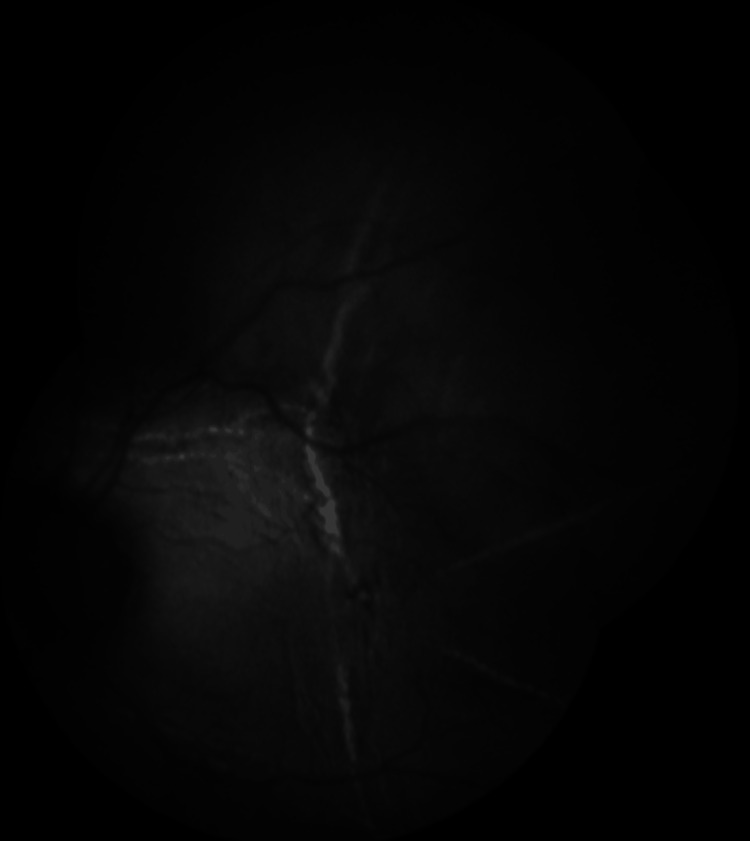
Fundus autofluorescence of the left eye after choroidal detachment remission

**Figure 6:**
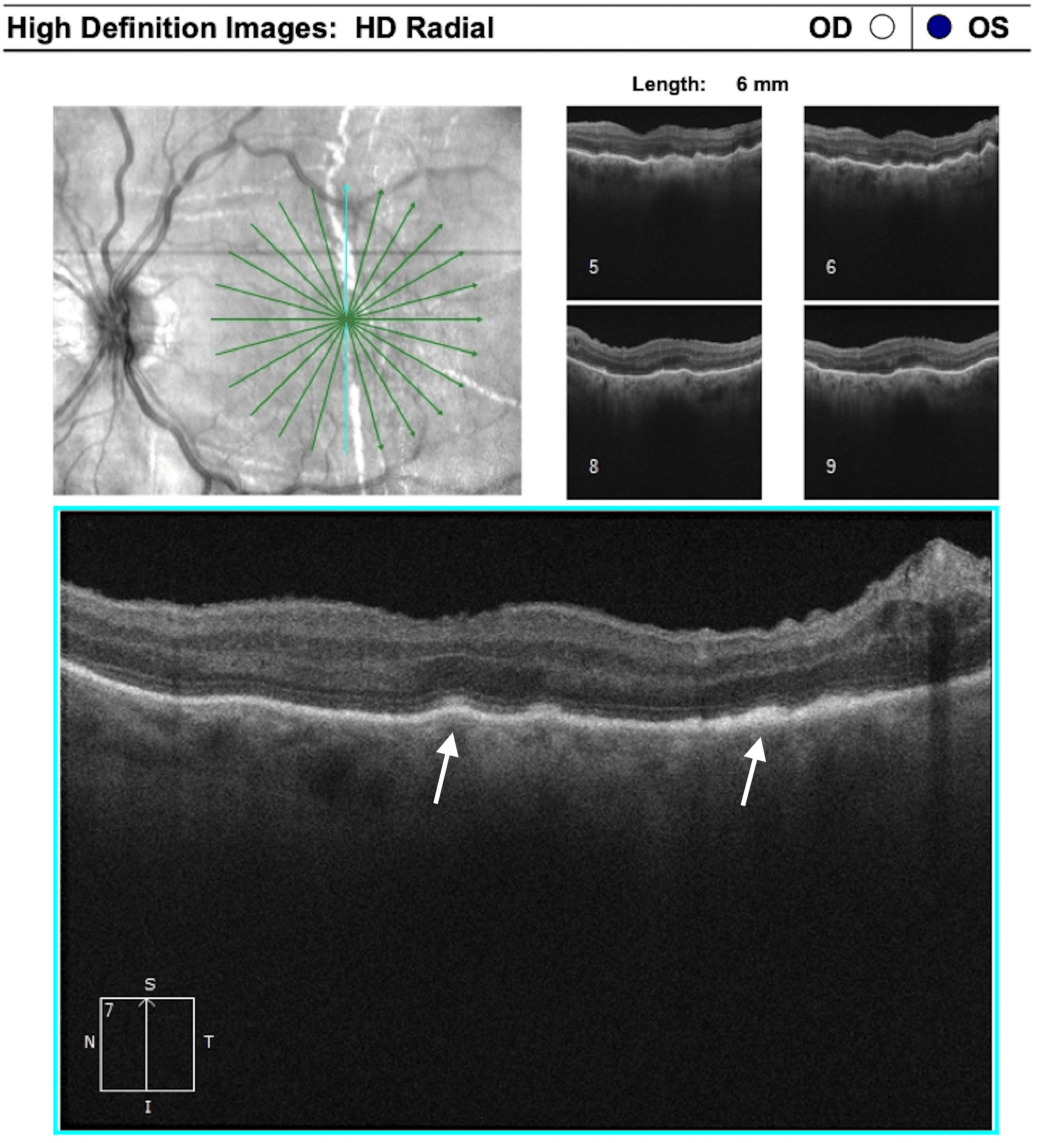
Optical coherence tomography after choroidal detachment remission showing retinal pigment epithelium hypertrophy (arrows) and persistent choroidal folds (panels 5 and 6, top right) OD: right eye, OS: left eye.

Even though the IOP increased to 22 mmHg and the choroidal detachment was resolved with intensive steroid and atropine administration in the following three weeks, the BCVA remained low (counting fingers at 2 m) 28 days after the drainage device placement. Fundoscopy revealed RPE changes extending beyond the macula toward the periphery and the optic nerve head, observed as dark and white lines (Figure 4). Importantly, autofluorescence clearly revealed the true extent of these hyperautofluorescent RPE lines radiating in a stellate pattern from the macula (Figure 5). High-definition optical coherence tomography demonstrated chorioretinal folds accompanied by RPE thickening at the areas corresponding to the RPE lines (Figure 6). Fluorescein angiography was not performed because the patient did not consent to intravenous fluorescein injection. 

**Figure 7:**
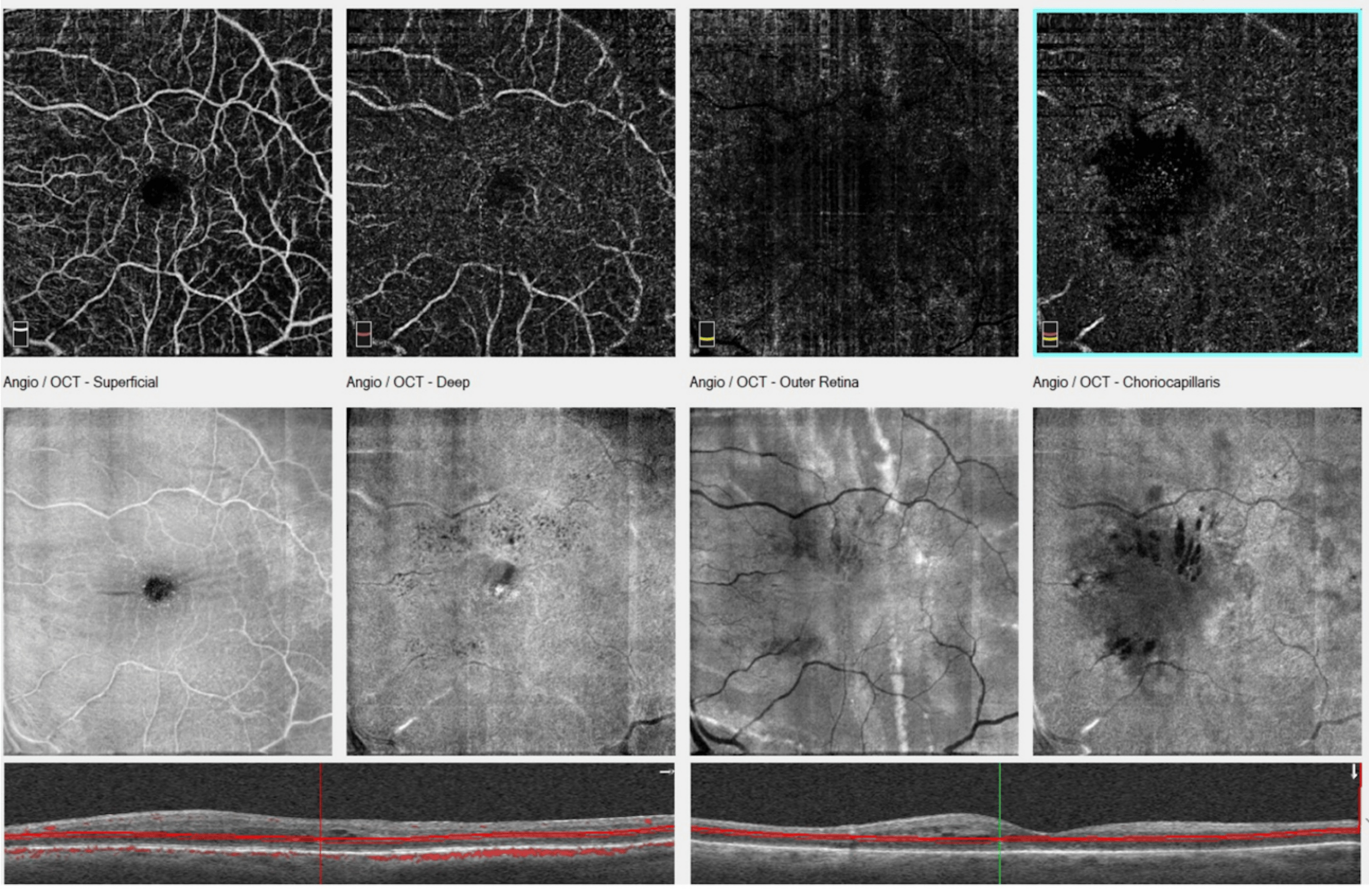
Optical coherence tomography (OCT) angiography and en face optical coherence tomography at six months of follow-up showing cystoid macular edema. Notice the appearance of the retinal pigment epithelium lines in the outer retina in en face optical coherence tomography imaging

These colored streaks appeared stable and did not change in size or shape over a six-month follow-up period. Visual acuity slightly increased to logMAR 1.0, and mild postoperative cystoid macular edema (CME) was observed in the six-month follow-up (Figure 7). The patient was prescribed topical nepafenac drops, and further follow-up was planned.

Table 1 shows the clinical course of the patient. 

**Table 1:** Clinical course of the patient BCVA: best corrected visual acuity, IOP: intraocular pressure, RPE: retinal pigment epithelium.

Time point	BCVA of the left eye (logMAR)	IOP of the left eye (mmHg)	Treatment
Initial presentation	0.40	54	Topical latanoprost and dorzolamide/timolol
One day before pars plana vitrectomy, and implantation of a retro-pupillary iris-claw lens	0.40	38	Topical latanoprost, dorzolamide/timolol, and dexamethasone, per os tab acetazolamide
One day after pars plana vitrectomy, and implantation of a retro-pupillary iris-claw lens	Hand movement	26	Topical latanoprost, dorzolamide/timolol, and dexamethasone, per os tab acetazolamide
One day before Paul implantation	0.70	40	Topical latanoprost, dorzolamide/timolol, per os tab acetazolamide
1 day after Paul implantation	Hand movement	2	Topical atropine and dexamethasone
7 days after Paul implantation/kissing choroidals observed	Hand movement	7	Topical atropine and dexamethasone
17 days after Paul implantation/resolution phase	Counting fingers at 2 m	12	Topical atropine and dexamethasone
28 days after Paul implantation/complete resolution of choroidals	Counting fingers at 2 m	22	Topical atropine and dexamethasone
5 months after Paul implantation/permanent RPE changes	1.00	14	No treatment
6 months after Paul implantation/cystoid macular edema	1.00	12	Topical nepafenac

## Discussion

This is a case in which unusual RPE changes were identified after choroidal detachment and hypotony maculopathy following the implantation of a Paul glaucoma drainage device. While early postoperative hypotony is a well-recognized complication of non-valved glaucoma drainage implants [1], the appearance of long-lasting or permanent RPE alterations of this type after a short hypotony period is barely described in the literature. It is estimated that hypotony after a Paul drainage device implantation ranges from 2% [5] to 35.4% [4]. This wide range may reflect differences in the surgical techniques, including the use of 6.0 intraluminal suture or strangulation sutures, which both appear to offer some protection against hypotony. Although postoperative hypotony is usually transient and self-limiting [4,5,9], in our case, persistent changes in the RPE were observed, despite the quick recovery of the IOP to higher values.

In 1996, Schubert [10] defined postoperative hypotony as low pressure (of about 5 mmHg), whether acute, temporary, or permanent, eventually leading to functional and structural changes in the affected eye. Two of these structural changes are choroidal detachment [10] and hypotony maculopathy [11]. The latter is characterized by chorioretinal folds caused by the collapse of the scleral wall, resulting in the redundancy of the choroidal and retinal layers and their wrinkling [8]. These changes are usually noticed around the fovea in a linear, parallel pattern and are the underlying cause of the alternating dark and light streaks in the posterior pole. In cases of prolonged hypotony, these streaks usually become more prominent, as they are associated with changes in the structure of RPE [8]. 

It is noteworthy that choroidal effusions rarely occur simultaneously with hypotony maculopathy, especially in the elderly, in whom the sclera is more rigid and less prone to collapse, and thus, transscleral ﬂuid outﬂow is expected to be reduced [7,12]. Interestingly, in such patients, the effusions could also play a protective role against the development of macular folds, as the suprachoroidal fluid that is accumulated helps maintain the scleral contour and counteract the sclera collapse during the hypotony period. However, this rare coexistence was indeed observed in our case, making it an exceptional finding.

Another noteworthy finding in the fundus of our patient is the unusual hypotony-related RPE changes that were observed just after the complete resolution of the kissing choroidal detachment. Their pattern, distribution, and thickness differ from what is usually observed, as the RPE changes extended radially beyond the macula, toward the temporal area, superiorly, and toward the optic nerve head without a uniform pattern. In addition, they were observed at the base and along the edges of the choroidal effusions, suggesting a topographical relationship between the area of detachment and the subsequent RPE remodeling. We postulate that the underlying mechanism is similar to that described in RPE changes caused by hypotony maculopathy. On the one hand, the mechanical stretching and the thinning of the RPE produce the light areas in the fundus, which appear as dark lines in the autofluorescence. On the contrary, the dark lines observed in fundoscopy correspond to the bright lines in autofluorescence and are probably the result of RPE thickening. As fundus autofluorescence can demonstrate the full extent of RPE lesions more clearly than color photography, it is a valuable tool when assessing cases with suspected RPE damage. 

There is no exact definition of long-term, chronic, or persistent hypotony. In most studies, it is defined as IOP ≤ 5-6 mmHg sustained over three months or more [13,14], and as described above, hypotony of this duration can cause permanent RPE damage [8]. However, it seems that duration is not the only important factor. The magnitude of hypotony, even over a shorter than three-month period, may be sufficient to cause RPE lesions, as in our case. 

As for the initial management, the definitive treatment of UGH syndrome includes IOL exchange, and in fact, the UGH represents one of the most common indications for IOL exchange [15]. In our case, the direct contact of the IOL-capsular bag complex with the iris led to UGH, which was likely secondary to zonular weakness associated with exfoliation syndrome. Although one might speculate that exchanging the lens for an iris-claw IOL, given its position close to the iris, could similarly provoke irritation, there is no robust evidence that iris-claw lenses should be avoided in UGH syndrome, as they are generally considered to be a safe option [16]. Despite the complete resolution of UGH-related signs after iris-claw IOL implantation, the IOP remained elevated and resistant to medical therapy, likely due to irreversible trabecular damage caused by high intraocular pressure and blood infiltration.

Last but not least, CME was observed in the affected eye of our patient in the last follow-up, six months after the drainage device implantation. It is well known that intraocular inflammation and surgery increase the risk of postoperative CME. This is particularly true in cases with capsular compromise, vitreous surgery, and/or iris tissue manipulation [17].

To the best of our knowledge, this case is perhaps the first of an adult patient in which this type of apparently permanent dark and light-colored hypotony-related RPE changes are described after a short period of hypotony complicated by choroidal effusion. In 2018, Osigian et al. [18] described similar hypotony-related RPE lesions in newborns, though without preceding choroidal detachments. In our case, the prior vitrectomy may have compromised the internal support of the vitreous body, thus allowing a greater mechanical stretch of the RPE at the edge of the choroidal detachment. Furthermore, this lack of internal support could also explain the simultaneous occurrence of both choroidal detachment and hypotony maculopathy, suggesting that a history of vitrectomy may be a risk factor for hypotony-related retinal complications. In such cases, early detection and management of hypotony might be beneficial, although this cannot be ascertained based on the currently available evidence.

## Conclusions

This case highlights the fact that even transient episodes of ocular hypotony complicated by choroidal detachment and hypotony maculopathy can lead to irreversible RPE damage. The location of these RPE changes is inextricably intertwined with the area of the choroidal detachment. In the unfortunate event of such RPE alterations involving the area close to or underneath the macula, permanent visual handicap might occur. Therefore, clinicians should maintain a high level of clinical suspicion for such RPE changes in cases where the visual acuity cannot be fully restored after the resolution of the hypotony-related complications. Notably, even in the absence of clinically prominent lesions following the hypotony resolution, autofluorescence can be particularly useful for the assessment of these subtle but vision-threatening RPE changes. The early recognition of hypotony and the appropriate adjustment of aqueous flow after glaucoma surgery may be important in order to avoid permanent damage to the RPE. However, more similar cases are needed to corroborate our observations.
